# Examining the Relationship between Mindfulness, Personality, and National Culture for Construction Safety

**DOI:** 10.3390/ijerph18094998

**Published:** 2021-05-08

**Authors:** Tomay Solomon, Behzad Esmaeili

**Affiliations:** Sid and Reva Dewberry Department of Civil, Environmental and Infrastructure Engineering, Volgenau School of Engineering, George Mason University, Fairfax, VA 22030, USA; tsolomo@gmu.edu

**Keywords:** mindfulness, personal characteristics, construction safety

## Abstract

The construction industry still leads the world as one of the sectors with the most work-related injuries and worker fatalities. Considering that one of the barriers to improving construction safety is its stressful working environment, which increases risk of inattentiveness among construction workers, safety managers seek practices to measure and enhance worker focus and reduce stress, such as mindfulness. Considering the important role of mindfulness in curbing frequency and severity of incidents, researchers are interested in understanding the relationship between mindfulness and other common, more static human characteristics. As a result, this study examines the relationship between mindfulness and such variables as personality and national culture in the context of construction safety. Collecting data from 155 participants, this study used elastic net regression to examine the influence of independent (i.e., personality and national culture) variables on the dependent (i.e., mindfulness) variable. To validate the results of the regression, 10-fold cross-validation was conducted. The results reveal that certain personality traits (e.g., conscientiousness, neuroticism, and agreeableness) and national cultural dimensions (e.g., uncertainty avoidance, individualism, and collectivism) can be used as predictors of mindfulness for individuals. Since mindfulness has shown to increase safety and work performance, safety managers can utilize these variables to identify at-risk workers so that additional safety training can be provided to enhance work performance and improve safety outcomes. The results of this study will inform future work into translating personal and mindfulness characteristics into factors that predict specific elements of unsafe human behaviors.

## 1. Introduction

With more than 1000 fatalities among construction workers every year in the United States alone [1] and nearly $6 billion costs in lost production, lost income, and pain and suffering [2], the safety performance of construction workers demands improvement. Improving construction safety is challenging since workers have to execute their tasks under both physically and psychologically demanding conditions [3,4] to meet time and budget constraints in a project. Such working conditions can create inattentiveness [5], anxiety [6], or stress [7,8,9], which contribute to construction workers’ unsafe behaviors [9]. Therefore, safety managers seek practices to enhance worker focus, reduce stress, promote caution, and hone workers’ abilities to identify, acknowledge, and respond to uncertainties in the workplace, ultimately reducing human errors leading to accidents [10]. 

One technique to lower stress and anxiety, enhance focus, and improve attentional performance is to implement mindfulness practices. In the last decade, an explosion of interest in mindfulness research on the concept and application of mindfulness has taken place, and mindfulness journal publications increased from fewer than 35 articles in the early 2000s to 1203 publications in 2019 (goAMRA.org (accessed on 8 March 2021)). Originating in Buddhist philosophy and meditation practice [11], mindfulness has been shown to be effective in treating mental and behavioral health issues [12,13]. Mindfulness as a trait is particularly related to attention and awareness [14], which are essential factors in workplace safety [15]. Because being mindful can help reduce workplace accidents and injuries, it is important to identify which individual differences, such as personality, influence the state of mindfulness of construction workers. This approach could potentially screen out accident-prone employees [16,17] who may need additional support or training to prevent accidents.

Considering the important role of mindfulness in curbing frequency and severity of incidents, researchers are interested in understanding the relationship between mindfulness and other common, more static human factors, such as personality [18,19]. Although recent research on personality shows that certain personality traits are highly related to safety and to the attentional failures that may lead to unsafe behaviors, e.g., Hasanzadeh et al. [17], and little is known about other personal factors that may impact mindfulness. More recent studies even have suggested that the relationship between personal characteristics and safety performance may be mediated by failures in cognitive processes, such as poor selective attention or distractibility [17]. Considering that mindfulness is particularly related to attention and awareness [14], understanding the relationship between mindfulness and other common, more static human factors, such as personality will increase our knowledge regarding the mediating role of attention in safety performance. For instance, to what extent can a variable such as national culture makes certain employees more risk-, injury-, and accident-prone? 

To address this knowledge gap, this study examines the relationship between mindfulness and variables such as personality and national culture in the context of construction safety. The results of this study offer solutions for reducing accidents in the construction industry by providing additional factors that can be used as a predictive index. Furthermore, the results may be used as inputs to design better safety training programs to enhance worker safety on job sites, which, in turn, will conceivably lead to better safety performance.

## 2. Literature Review

### 2.1. Mindfulness

The original term commonly referred to as mindfulness is “sati”, a Sanskrit word that indicates both awareness and remembrance or memory [11]. While mindfulness stems from the approximately 2500-year-old Buddhist tradition, in its modern approach, mindfulness can be defined as “paying attention in a particular way, on purpose, in the present moment, and non-judgmentally” [20] (p. 4). Even though differences exist among experts on how mindfulness is defined and conceptualized [21], attention and awareness are the fundamental and common elements that make up mindfulness. 

In practice, mindfulness has been applied as a treatment to mental and behavioral health issues, including stress, anxiety, and depression [12,13,22,23], which incidentally can enhance workplace safety. Considering a great deal of stress caused by the working environment and the immense pressure to execute a job contributes to unsafe worker behaviors [9], and implementing mindfulness practices that can help workers be more attentive and aware of themselves and their surroundings has recently gained traction within industry [15,24,25]. In the following section, the research team will summarize the salient results of a literature review about mindfulness measures and the relationship between mindfulness and safety performance. 

#### 2.1.1. Measuring Mindfulness

Due to the existence of different mindfulness definitions, it can easily be inferred that different mindfulness measures exist. These measures differ from each other according to the total number of items and the respective dimensions they measure and also whether they consider mindfulness a trait or a state. Some of the most frequently used mindfulness measures are the Mindfulness Attention and Awareness Scale (MAAS) [14], the Five Facets Mindfulness Questionnaire (FFMQ) [26], the Kentucky Inventory of Mindfulness Skills (KIMS) [27], the Philadelphia Mindfulness Scale (PHLMS) [28], the Revised Cognitive and Affective Mindfulness Scale (CAMS-R) [29], the Freiburg Mindfulness Inventory (FMI) [30], the Southampton Mindfulness Questionnaire (SMQ) [31], and the Toronto Mindfulness Scale (TMS) [32]. 

In order to clarify which mindfulness measure to choose from among the validated mindfulness questionnaires, Qu et al. [33] evaluated eight frequently used validated measures of mindfulness. Using five evaluation techniques—namely, operational definition, content validity, high reliability, construct validity, and high criterion-related validity—they compared mindfulness measures by giving them a grade of high, moderate, low, or none. The results of their study showed that only the MAAS scored “High” on every evaluation measure. 

Developed by [14], the MAAS is a fifteen-item scale measuring mindfulness as a single factor relating to attention. The MAAS is designed to measure a conceptualization of mindfulness as “the presence or absence of attention to and awareness of, what is occurring in the present moment” [14] (p. 824). With the goal to further validate the MAAS as a reliable measure of mindfulness, MacKillop et al. [34] conducted a confirmatory factor analysis to compare the differences in meditation practice among participants in a large sample and concluded that the MAAS is a valid measure of mindfulness. In addition, this measure of mindfulness showed satisfactory psychometric properties and validity inferences [35,36]. Considering that MAAS has been used successfully by other researchers to measure the relationship between mindfulness and workplace safety in the healthcare industry, Dierynck et al. [37] and petroleum-distribution industry, Kao et al. [25], the authors decided to use this measure for the present study. 

#### 2.1.2. Mindfulness and Safety Performance

Previous studies have shown that mindfulness can have a positive impact on the safety performance of workers [10,15,38,39,40,41]. For example, Zhang et al. [39] investigated the influence of mindfulness on task complexity and safety performance among nuclear power-plant operators and found that mindfulness interacted with task complexity (significantly positive influence on both task and safety performance for high-complexity activities) to influence safety performance. In a follow-up study, Zhang and Wu [40] investigated the relationship between dispositional mindfulness and workplace safety on a sample of nuclear power-plant control-room operators and determined that mindfulness has a positive impact on workers safety compliance and safety behavior.

In a study examining dispositional mindfulness and its relationship with safety practices in the food industry, [42] found that mindfulness can be used as a predictor of both safety practices and safety knowledge. In addition, using moderation analysis, they found that mindfulness predicts safety practices better among workers with the least safety knowledge. In another study, Dierynck et al. [37] investigated the role of individual and collective mindfulness of nurses on self-reported workaround (short-cuts) rates and safety failures. The study found that both individual and collective mindfulness were significantly negatively correlated with workarounds, and the number of occupational safety failures were significantly positively correlated. As a result, Dierynck et al. [37] claimed that mindfulness has a positive effect on occupational safety in hospitals. More recently, [25] evaluated the relationship between the mindfulness trait and workplace injuries in the petroleum-distribution industry. Collecting and analyzing hierarchically nested data, they found that mindfulness is related to workplace injuries, safety compliance, and safety participation, and they observed this relationship is mediated by safety compliance. They concluded that the mindfulness trait is an important factor in determining safety behavior and subsequently in reducing the frequency of incidents.

In the construction industry, a limited number of studies have investigated the role of mindfulness in incident occurrence or safety behavior [10,15,43,44,45]. Considering that stress can negatively impact the performance of construction professionals, some of these studies have focused on the application of mindfulness in reducing stress at construction sites. For example, Liang and Leung [43] investigated the relationship between mindfulness characteristics and different kinds of stress experienced by construction professionals. They found that certain types of mindfulness characteristics can release or exacerbate different kinds of stress. In a follow-up study, Leung et al. [44] found that mindfulness characteristics indirectly improve construction workers’ performance by relieving their stress and directly improves safety performance through increased awareness. One of the interesting findings of the study was that decentering—or the ability of someone to be aware of his/her own experience but see it from another view—can harm safety performance.

While previous studies have advanced knowledge regarding the role of mindfulness in safety performance, little is known regarding the variables that impact the mindfulness state of construction workers. This study addresses this knowledge gap by investigating the impact of personality, national culture, and attitude on the mindfulness state of construction workers.

### 2.2. Personality

Though numerous studies have shown that personality traits can be used as a predictor for human behavior, e.g., [46], contributions from studies on personality factors, e.g., [47,48,49] produced a five-factor model—known as the “Big Five”—that continues to be the most widely accepted theory of personality today. The five dimensions of personality, as compiled by [50], include (1) *extraversion* (talkative, assertive, active, energetic, and outgoing), (2) *agreeableness* (sympathetic, kind, appreciative, affectionate, and trustful), (3) *conscientiousness* (organized, thorough, efficient, responsible, and dependable), (4) *neuroticism* (tense, anxious, nervous, moody, and worrying), and (5) *culture or openness* (imaginative, intelligent, original, insightful, and curious). These factors provide an effective and quantifiable metric for gauging individuals’ personality traits, which has thereby enabled research on the effects of personality in a breadth of sectors.

The relationship between personality and mindfulness has been studied in more detail by researchers, e.g., [18,51]. A meta-analysis on 29 studies that included 32 independent samples conducted by Giluk [18] found that the personality trait neuroticism was negatively associated with dispositional mindfulness, and conscientiousness and agreeableness were positively associated. Giluk’s meta-analysis also found a weak positive relationship between extraversion and openness with dispositional mindfulness. These results were also reinforced by Tucker et al. [51], with neuroticism being negatively associated with mindfulness while positive associations existed with the other dimensions of personality. A canonical correlation analysis conducted by Hanley [52] also found that the strongest relationship with mindfulness was between neuroticism (negatively associated) and conscientiousness (positively associated).

The potential links between personality and mindfulness can be further exploited to examine the work and safety performance of workers. Since individual personality traits do not change much over time, the personality of individuals can be used as a tool to predict the dispositional mindfulness of workers.

### 2.3. National Culture

One variable that impacts attention [53] and safety behavior [54] is national culture, a factor that becomes more salient considering the increasing workforce diversity in the construction sector [55,56,57,58,59]. Numerous cross-cultural studies show that culture has an effect on risk-taking behavior [54,60], risk perception and understanding [61], following orders and procedures [62], level of safety [63,64,65], perception of acceptable levels of safety [66], and cognitive style of information processing and decision making [67].

Culture has been defined as shared experience, beliefs, values, attitudes, religion, and conception of the world gathered during lifetime of a person that is passed to future generations [68]. Considering the complexity and elusiveness of culture, researchers have made numerous attempts to break culture into its fundamental constructs. One of the most successful attempts to dimensionalize culture—initiated and expanded by Hofstede [68,69,70]—resulted in the following dimensions (Table 1): (i) power distance, (ii) uncertainty avoidance, (iii) individualism and collectivism, (iv) masculinity and femininity, and (v) long-term orientation.

#### 2.3.1. Measuring Culture at the Individual Level

One of the main criticisms about Hofstede’s cultural model concerns about its limitation in measuring cultural dimensions at the individual level [68,71,72]. Accordingly, [73] highlighted the importance of individual-level analysis in determining “relationships among organizational variables that are sensitive to certain cultural differences.” They stated that Hofstede’s ecological meaningfulness embedded ambiguity, and they raised the limitation of the model’s efficiency at micro-level analysis.

Anthropologists were among the first group of researchers who addressed this knowledge gap by developing frameworks to measure cultural dimensions at the individual level [74]. In a more recent study, investigating the isomorphism of individual and country levels of cultural value constructs, Fischer et al. [75] demonstrated that individualism index of cultural dimension could be utilized at the individual level. Based on the work of Hofstede’s cultural dimensions, Yoo et al. [72] developed a survey, the Individual Cultural Value Scale (CVSCALE), capable of measuring an individual’s cultural indices to address the deficiencies of Hofstede’s cultural questionnaire. Yoo et al. [72] tested the reliability of the CVSCALE questionnaire and concluded that the scale resulted in reliable cultural dimension and invariant factor loadings and can be used for cross-cultural comparisons. Subsequent studies on individual cultural dimensions have utilized this scale in their research [76,77,78,79,80,81,82]. In a multicultural study with two independent samples (*n* > 500) comprising immigrant ancestors (no Native Americans) population, [83] applied the CVSCALE and concluded that this scale can be used to reliably measure national culture dimensions at the individual level or psychological level. In this study, the CVSCALE is used to measure national culture at the individual level.

#### 2.3.2. Role of National Culture in Construction Safety

The role of national culture in dissimilar safety performance has been studied in the construction industry, e.g., [66,84,85]. One of the earliest studies that used Hofstede’s national culture dimensions was conducted by Mohamed et al. [54], who examined the correlation between construction workers’ behavior, attitude, and perception towards safety with the safety culture in Pakistan. Using factor analysis to combine some of the national culture dimensions, the study’s authors identified three main factors: collectivism and femininity (48% of total variance), uncertainty avoidance (18% of total variance), and power distance (14% of total variance). Using the correlation analysis, they found that workers with higher uncertainty avoidance tend to be more safety aware and had a stronger belief in safety issues, and as the power distance between workers and management grows, workers will have lower awareness and beliefs regarding safety issues. Then, Ref. [54] conducted a binary logistic regression analysis to assess the independent effects of each of the dependent variables (i.e., attitude, perception, and cultural dimension) on different behavioral situations. The results showed that workers with higher collectivism, femininity, and uncertainty avoidance are more likely to avoid continuing to work under risky situations. The cultural dimension of power distance could not predict any of the given behavioral situations.

To empirically measure the impact of individual cultural values on the risk perception of construction workers, Habibnezhad and Esmaeili [82] measured both the cultural dimensions and risk perception of construction workers using a questionnaire. The findings showed that workers with higher uncertainty avoidance and collectivism selected lower probabilities for low-impact consequences (e.g., first aid or medical case), especially for fall hazards. In contrast, those with a larger masculinity index assign lower probabilities to high-impact consequences (e.g., fatality) compared to those with lower masculinity.

Al-Bayati and his colleagues [57,58] used multiple techniques—including questionnaires, interviews, and focus groups—to better understand the existing cultural differences and their influence on safety performance among Hispanic workers. By considering culture as a guiding principle that may influence one’s behavior given the social environment [70], Al-Bayati et al. focused on identifying cultural differences that influence safety performance of construction workers. Collecting and analyzing perspectives of both Hispanic workers and their supervisors, Al-Bayati et al. could identify three active cultural differences [57,58]: high power distance, collectivism, and uncertainty avoidance. The findings show that, due to higher power distance, when the task is unsafe, it is more common for Hispanic workers not to object or deliver their concerns to their supervisor. In addition, since Hispanic workers typically work with their family members or close friends, they tend to trust their fellow Hispanic coworkers or supervisors more than others. Finally, due to higher uncertainty avoidance, Hispanic workers preferred detailed instructions to successfully complete a task.

In summary, the literature review shows that even though personality traits and mindfulness have shown relations, the effect of national culture of workers in combination with personality on mindfulness is still unknown. To address this knowledge gap, this study investigates the degree of association of mindfulness with relatively static factors including personality traits and national culture to predict the mindfulness of individuals.

## 3. Materials and Methods

In order to examine the influence of the independent (i.e., personality and national culture) variables on the dependent (i.e., mindfulness) variable, this study used a regression analysis. In this section, the authors detail the study’s data collection instruments, participants characteristics, data analysis approach, and validation.

### 3.1. Data Collection Instruments

#### 3.1.1. Dependent Variable

To measure mindfulness, this study used the Mindfulness Attention Awareness Scale (MAAS). Developed by Brown and Ryan [14], and regarded as one of the most established techniques for measuring mindfulness [86], the MAAS applies a Likert scale ranging from 1 to 6 (almost always to almost never), and participants must respond to 15-item statements in the questionnaire. The average of all statements was reported as the participant’s mindfulness score, with higher scores indicating a higher level of dispositional mindfulness and vice versa.

#### 3.1.2. Independent Variables

To assess personality, this study used the Big Five Inventory (BFI) developed by [50]. This 44-item questionnaire also uses a Likert scale ranging from 1 to 5 (strongly disagree to strongly agree). Like any Likert-based questionnaire, participants answer to which extent they agree or disagree with each statement. Total scores are then calculated by adding the direct and reverse score Likert value to each of the personality types, as specified by [50].

To compute the national culture aspect of participants, this study utilized a questionnaire adapted from [54]. National culture has four dimensions: *power distance* (five items), *individualism* vs. *collectivism* (six items), *uncertainty avoidance* (five items), *masculinity* vs. *femininity* (four items). National culture was also computed based on a Likert scale like personality. The scale ranges from 1 to 5 (strongly disagree to strongly agree) for national culture.

### 3.2. Participants

A total of 156 participants (30 construction workers and 126 students) aged 18–62 years (mean = 26.81, standard deviation = 9.55) were recruited to provide the data; however, one data point was removed from the student sample because it was incomplete. Therefore, a total of 155 participants (30 construction workers and 125 students) were considered for analysis. The student respondents were from the department of civil engineering at George Mason University. Since previous studies have shown that experience plays an important role in determining safety performance of people involved in construction activities [87,88], student sample data were divided into two groups: students with experience and novice students. The students with experience sample were between 21 and 40 years old (mean = 24.42, standard deviation = 4.46), novice student sample were between 18 and 40 years old (mean = 23.44, standard deviation = 4.33), and the construction workers’ sample were between the ages of 19 and 62 years old (mean = 39.69, standard deviation = 13.89). Participants were recruited through on-campus fliers, posting an invitation flyer at construction sites, and stopping by construction companies’ main offices. All participants provided written informed consent, and workers were given $15 gift cards, whereas students received classroom credit points as compensation after finishing the questionnaires. All procedures were approved by the Institutional Review Board (IRB) of George Mason University.

### 3.3. Data Analysis Approach

In order to analyze data that contains multiple independent variables, generalized regression approaches are appropriate to select which variables have the most significant effect on the response variable. Before conducting any regression analysis, the assumptions for regression should be tested. Therefore, the research team first tested the distribution of the data and the existence of potential outliers. The assumptions for regression were checked for each dataset separately (i.e., students with experience, novice students, and workers). Plotting the distribution of the mindfulness score for construction workers, it was found that the data were normally distribution with no outliers (Shapiro–Wilk: *p*-value 0.73 > 0.05). For novice student data, one data point was an outlier (same data point as the outlier) and removed from data in the analysis. By removing the outlier, the distribution of the dependent variable changed from non-normal (Shapiro–Wilk: *p*-value 0.01 < 0.05) to normal (Shapiro–Wilk: *p*-value 0.07 > 0.05). The mindfulness score of students with experience was normally distributed with no outliers (Shapiro–Wilk: *p*-value 0.09 > 0.05).

Then, to confirm that the relationship between the independent variables and the response is linear, the research team evaluated the scatter plot of each independent variable with the response variable. There was no pattern to show violation of linearity, such as curvilinear or cubic, on the dataset. To address the assumption regarding the variance of the residuals of the response variable, the authors plotted the residuals versus predicted values and observed no pattern for the datasets. All observations proved to be independent of each other, and finally, the distribution of the residuals of the dependent variable were normally distributed for students with experience (Shapiro–Wilk *p* = 0.97) as well as construction workers (Shapiro–Wilk *p* = 0.17). However, the distribution of the residuals of the dependent variable were not normally distributed for novice students (Shapiro–Wilk *p* = 0.003). Plotting the distribution of the residual for novice students revealed that one data point was an outlier and removed from the analysis. After removing this data point, the distribution of the residuals of the dependent variable changed from non-normal (Shapiro–Wilk: *p*-value 0.003 < 0.05) to normal (Shapiro–Wilk: *p*-value 0.117 > 0.05).

In any regression approach, the first starting point to do any type of regression is linear regression. Simple linear regression, also known as ordinary least squares (OLS), attempts to minimize the sum of error squared. Even though this regression method is key to understanding the nature of the regression model, it usually oversimplifies the analysis and thus important variables may not be selected as they should be. The other disadvantage of linear regression is that it is prone to multicollinearity, which means that if there is high correlation between the predictors, removal of one or more variables from the analysis may be necessary, which is problematic because those variables may be important predictors for the response variable at hand. Hence, a more refined type of approach is needed to better select variables while also simplifying the model by retaining the significant variables and removing the variables that do not contribute to the prediction of the response variable.

### 3.4. Penalization Methods

Traditional regression methods such as stepwise, forward, and backward selection suffer from high variability and low prediction accuracy, especially when there is a correlation between variables or multiple predictors [89,90]. In response to these shortcomings, using penalized regression methods have gained traction among researchers due to their higher prediction accuracy and computational efficiency [91]. Using penalized estimates in a regression model, the user accepts some bias in order to reduce variance. Similar to ordinary least squares (OLS) estimation, penalized regression methods estimate the regression coefficients of the predictors by minimizing the sum of squares of the residuals; however, in contrast to OLS methods, the penalized regression places a constraint or penalty, e.g., [92] on the size of the regression coefficients, which causes the coefficient estimates to be biased. The introduction of the penalty improves the prediction capability of the model by decreasing the variance of the coefficient estimates.

Generalized regressions with no penalties are based on the least square estimation method, which is an unpenalized fit and provides no simplification (no variable selection) and no shrinkage of parameters. Alternatively, penalized regression selects variables by minimizing the sum of the squared residuals while also adding a penalty proportional to the size of the regression coefficients. If the size of the penalty on a specific parameter is large enough, it causes the regression coefficient to shrink towards zero. Hence, some variables will be removed from the analysis, which will simplify the final model by selecting fewer variables. In the same token, shrinkage is done by continuously shrinking the regression coefficients by introducing some degree of bias, e.g., [92,93,94] in the coefficient estimates. More often, the introduction of bias tends to reduce variance, resulting in a model with a better prediction performance.

In this study, we utilized a type of generalized penalized regression called elastic net regression, which can select variables by introducing a penalty in the regression model. In many statistical models, the typical technique behind the penalized regression analysis is using an estimation method called maximum likelihood. This estimation method delivers the best fit based on the observed data. By applying a penalized likelihood instead, better prediction on the response variable can be achieved.

The purpose of introducing a penalty is to achieve two main purposes. The first purpose is to allow the model to perform variable selection by removing unimportant predictors, and the second purpose is to apply shrinkage of estimation parameters. By optimizing the penalized likelihood, the regression model is simplified (fewer predictors), overfitting (weak prediction performance) can be avoided, and issues that arise from multicollinearity (high correlation between predictors) can be resolved [95]. In other words, by applying penalized estimates in regression, some degree of bias is accepted in order to reduce variance. The penalization regression methods and corresponding penalties are shown in Table 2.

In ridge regression, the coefficients on the predictors are shrunk by imposing a penalty (i.e., β_j_^2^)—also written as L_2_ penalty—such that the ridge coefficients minimize a penalized sum of residual squares. The disadvantage of the ridge regression is that it shrinks the coefficients to non-zero values to prevent overfitting and keeps all the variables. Hence, this approach is not a viable option to reduce variable selection.

As with ridge regression, Lasso regression has a shrinkage approach but with a subtle difference: The L_2_-penalty is replaced by L_1_-penalty (i.e., |β_j_|). This method shrinks the less important variable coefficients to zero and therefore can remove the variables that are deemed not significant predictors for the response variable. Even though Lasso both shrinks and selects by removing variables, studies have shown that Lasso tends to yield a model that is more parsimonious (less complex) than the elastic net approach. The other shortcoming of this method is that in the case of collinearity, the Lasso model selects the variable with the strongest correlation with the response variable and drops the other variables from the model.

In this study, we selected to use the elastic net approach for our data analysis. This method was proposed by [92] and utilizes an algorithm called LARS-EN, which was adapted from LARS for Lasso [96]. This method also uses both ridge and Lasso regression penalties and combines the techniques from the two methods by learning from their limitations to improve on the regularization of the model. Elastic net regression can be written as follows:

(1) $$\mathrm{Sum}\;\mathrm{of}\;\mathrm{squared}\;\mathrm{estimate}\;\mathrm{of}\;\mathrm{errors}\;{\left(\mathrm{SSE}\right)}_{\mathrm{Elastic}\;\mathrm{net}\;}=\sum_{i=1}^{n}(y_{i}-\beta o\;-\sum_{j=1}^{p}X_{ij}\beta_{j}{)}^{2}\;+\;\lambda \sum_{j=1}^{p}(\left(1-\alpha \right)\;{\beta_{j}}^{2}+\alpha |\beta_{j}|)$$

where X = (x_1_, x_2_, …. x_p_) are input variables, α is the alpha parameter, λ complexity parameter and y is the response variable. As can be seen from the above equation, when alpha is zero, the regression equation becomes ridge regression, and when alpha is one, the equation becomes Lasso.

The general mechanics of the elastic net is executed in two steps. First, the algorithm finds the ridge regression coefficient and on the second step uses a Lasso-sort of shrinkage of the coefficients. To eliminate the limitations found in Lasso, the elastic net includes a quadratic section of the penalty, which increases variable selection. It is worth noting that the quadratic section of the penalty (i.e., (1−α) β_j_^2^) when used in isolation (α = 0), becomes ridge regression. The other advantage of elastic net regression is grouping. If there is a very high correlation among independent variables, then this method performs well in incorporating variables into the model that aids in better prediction accuracy, unlike the Lasso approach, which tends to select only one variable from the highly correlated independent variables. In addition, simulation studies done on real-world data have shown that the elastic net approach often performs better than Lasso [92].

### 3.5. Validation

Cross-validation technique utilizes different samples of data to increase the overall accuracy of the predictive model [97]. In this study, k-fold cross-validation is used as it is the most widely used method for estimating prediction error [94]. The choice of k is usually 5 or 10, but there is no hard and fast rule [98]. For a relatively small dataset (as the case here), k = 10 is chosen and therefore used in the place of k, yielding the 10-fold cross-validation. In 10-fold cross-validation, training data are randomly broken into 10 groups or folds of approximately equal sizes. The first part or fold is used for the validation set and the rest of the data are fit for the remaining folds. This is repeated 10 times with a different part used for error estimation.

## 4. Results

The descriptive statistics of variables studied are presented in Table 3. On average, workers have higher mindfulness, power distance, and conscientiousness scores. Average scores for extraversion and openness are almost the same for students with experience, novice students, and workers data sets. Looking at the standard deviation (SD) columns, both the student samples have a much higher neurotic SD than workers’ sample. The workers’ SD is higher for conscientiousness as compared to the students with experience and novice students’ sample.

The correlation matrix between all variables considered in the analysis for the students with experience data (ES), novice students (NS), and for the construction workers’ data (W) appears in Table 4. There were no issues with multicollinearity (i.e., correlation greater than 0.7) between independent variables (see Table 4). In addition, the Cronbach alpha of personality, national culture and mindfulness are computed, and all the items are greater than 0.7 (see Table 5). To test whether the common method bias exists in the collected data, the research team also conducted Harman’s single factor test. Since the cumulative percent of variance was 20.85% (less than 50%), the impact of common method bias was not a substantial threat.

As described above, the research team applied the adaptive elastic net method of estimation to select the independent variables that are significant predictors of mindfulness score. The adaptive estimation utilizes a modified version of the L_1_ penalty via weights generated from the maximum likelihood estimates of the predictors to improve the overall fit of the model. K-fold (10-fold) cross-validation compared the training set with the testing set of the data. As the alpha value can be a value within zero and one (α ∈ (0, 1)), the model can move between ridge (α = 0) and Lasso regression (α =1). To select an α-alpha value to be used in the elastic net regression, a set of values (0.10, 0.25, 0.5, 0.75, and 0.90) were compared using a standard error value and the best alpha value was selected for fine tuning. Table 6 shows the different alpha values and the corresponding errors. Alpha values of 0.5, 0.25, and 0.75 were chosen to be used in the elastic regression with the least error for students with experience, novice students, and workers datasets, respectively.

The results of the adaptive elastic net regression with 10-fold cross-validation appear in Table 7. As one can see, the variance inflation factor (VIF) values are less than five, assuring no collinearity between independent variables. On the estimate column, the elastic net regression model equates to zero for some of the independent variables. This means the model has removed the variables from the analysis by shrinking the coefficient all the way to zero and selected fewer variables that explain the response variable. The negative sign of the estimates depicts an opposite relation between the predictors and the response variable. Higher *agreeableness* scores positively correlate with mindfulness among novice students, and higher *neuroticism* scores negatively correlate with mindfulness for both student samples. For workers, the significant predictors are *uncertainty avoidance* and *conscientiousness*, which all show significant positive correlation with mindfulness score.

The Wald test (also called the Wald Chi-Squared Test) is a test that examines whether explanatory variables in a model are significant. For variable estimates that are non-zero, the Wald test is calculated for significance, and the corresponding *p*-value is computed. The lower 95% and upper 95% show confidence interval (CI) ranges at 0.05 significance. CIs less than zero signify a negative relationship of predictors with the response variable, and positive relationships appear in CI ranges greater than zero. The corresponding *p*-values for Wald statistic for the estimates of each variable are used to select the predicator variables if the *p*-values are found to be significant (i.e., *p* <0.05).

The variables included in the equation are significant predictors with negative coefficients, showing negative direction with the response variable (mindfulness) and vice versa with positive coefficients. The prediction expression for the dependent variable mindfulness for students with experience, novice students, and workers can be written as Equations (2) to (4), respectively:

MAAS _Students with experience_ = 5.38 − 0.08 × Neuroticism − 0.24 × Individualism vs. collectivism (2)

MAAS _Novice students_ = 4.00 + 0.03 × Agreeableness − 0.04 × Neuroticism (3)

MAAS _Worker_ = −0.01 + 0.10 × Conscientiousness + 0.34 × Uncertainty avoidance. (4)

Our data show that for construction workers, conscientiousness personality trait, and uncertainty avoidance of national culture dimensions all positively correlate with mindfulness. Uncertainty avoidance of the culture dimension (i.e., the largest estimator) is associated with minimizing taking risks, and individual that have high uncertainty avoidance scores are likely to give importance to have instructions that are detailed and closely follow instructions and procedures for standardized work. This signifies that by avoiding uncertainty, workers are mindful of not taking short cuts that undermine their safety. The higher conscientiousness signifies higher mindfulness score because mindfulness is associated with higher awareness of their surroundings. Therefore, these variables are important indicators of mindfulness among workers, and lower measure of these variables can imply lower mindfulness and consequently lower safety performance.

Within the student data, agreeableness positively affects mindfulness for novice students, while neuroticism was found to be negatively associated with mindfulness for both students with experience and novice students. A higher degree of agreeableness shows that individuals can go along with the people around them and are less combative in nature, which is related to non-judgmental attitude of mindfulness. Neuroticism trait of personality is associated with anxiety and stress, which negatively impact mindfulness of individuals. The results confirm the negative association of this personality trait with mindfulness, and higher value of neuroticism scores can be used as a predictor of lower mindfulness. With respect to cultural dimensions, for students with experience, higher individualism was associated with lower mindfulness. This could be because students with experience are much younger and possibly more individualistic than workers; however, in construction sites, workers become more risk averse due to the existence of hazards and care more about their co-worker’s safety.

## 5. Discussion

Mindfulness has shown to improve cognitive processes, such as attention, e.g., [99], which is an important factor in hazard identification and ultimately safe behavior in a construction site. By measuring the impact of personal characteristics on mindfulness as an indicator of attention, construction supervisors can identify at-risk workers that should receive personalized training or assigned to less cognitive-demand activities. Results from elastic net regression show that certain aspects of personality traits, and national culture dimensions affect the mindfulness score of participants. The results of the analysis and their relationship with past literature are discussed here for students with experience, novice students and workers.

### 5.1. Personality

#### 5.1.1. Neuroticism

Analyzing the data collected from students with experience and novice students here show that neuroticism is significantly negatively related with mindfulness. This result confirms the outcome of previous studies outside of construction safety field that have consistently found that neuroticism is negatively correlated with mindfulness [18,19]. This finding can be explained with the fact that neurotic personality has been associated with psychological distress, being distracted and lower psychological well-being [100]. Studies have also shown that individuals who have higher neurotic scores tend to struggle with stress [101], which is also negatively related to mindfulness. As compared to people with higher neurotic scores, mindful people have been associated with self-regulation of thoughts, and mental and psychological well-being [13]. In fact, mindfulness practices have repeatedly shown positive results in stress reduction [102,103,104].

The fast paced, demanding, and sometimes dangerous environment of construction sites create stressful conditions for individuals [7,105] and individuals who exhibit higher neurotic personality are likely to be less mindful (Table 7). Being less mindful in a work environment, such as a construction site, can put individuals in harm’s way. Investigating the relationship between neuroticism and accident involvement in the construction industry [106] found a high correlation between the neuroticism trait score and the degree and number of recorded injuries. In another study, Hasandazeh et al. [5] measured the attentional distribution of workers using a mobile eye-tracking apparatus and compared the differences in attentional allocation and situational awareness between workers with high and low neurotic scores when exposed to fall-to-same-level hazardous situation. Results of Hasandazeh et al. experiment show that less neurotic workers are better aware of their surroundings and are more attentive to the associated tripping hazards. In other words, safety performance of individuals can be negatively affected for individuals who have dominant neurotic personality trait. The findings of these studies further highlight the significant role of neuroticism trait in increasing accident involvement of construction workers. In addition, measuring this personality dimensions of individuals can be used as a predictor variable to estimate their dispositional mindfulness, which is highly related with work performance on the job [38]. To prevent potential future incidents, safety managers should devout more resources in terms of training or mindfulness practices to workers with higher neurotic scores.

#### 5.1.2. Agreeableness

The results of this study also show a positive relationship between the agreeable personality trait of novice students with mindfulness. Such a finding coincides with past work that shows agreeable individuals are generally sociable, cooperative, caring, and supportive of others [107]. In addition, people who are aggregable by nature tend to have high interpersonal skills and perform very well with other individuals. They also tend to be less combative and avoid altercation with others. These characteristics of individuals are related to empathy towards others, which is an important aspect of mindfulness [108]. Since in a construction site, teamwork and communication are necessary to accomplish project goals [109], agreeable individuals tend to better understand the challenges other people face in a work environment and be more mindful. Previous studies have also shown that individuals who have high agreeable scores tend to have better safety attitude and consequently are involved in fewer accident on the job [16,110].

#### 5.1.3. Conscientiousness

The conscientiousness of construction workers in this study was found to be significantly positively associated with mindfulness. These findings are consistent with the positive role of conscientiousness with safety performance of individuals [111] as this personality trait is associated with self-regulation, where the attention of individuals is highly increased [38]. Being attentive on a task-oriented work environment such as construction is vital for detecting and responding to hazards. Low scores of conscientious personality trait can be a predictor of lower mindfulness. Since low mindfulness is an indicator of lower attention and situational awareness, measuring personality traits related to lower mindfulness provides an indirect measure in detecting inattentive workers.

Conscientious individuals are usually careful, reliable, dependable, and goal oriented [112], and this personality trait is shown to be highly (positively) related to mindfulness [18,51,52,113]. Previous studies even suggested similarities between mindfulness and conscientiousness, as both are characterized by responding rather than reacting to stimuli [22]. Conscientious individuals have great self-regulation, which is one of the elements of being present/attentive, a major component of being mindful that can enhance safety performance on the job site.

### 5.2. National Culture

#### 5.2.1. Individualism vs. Collectivism

This dimension of national culture was negatively correlated with the mindfulness score for the students with experience sample. Individuals who are characterized with high individualism usually are on their own and have opinions and beliefs independent from the group, whereas low individualism people give opinions as a group, tend to work together, and give high regard to harmonious working relationship [54]. Mindfulness is associated with empathy, care, and understanding for others [14,114]. One reason that this cultural dimension was so prevalent and negatively related to mindfulness can be explained with the fact that students who are individualistic care less about their counterparts and will have less mindfulness measure. The mean age difference between students with experience (24.42 years) and construction workers (39.7 years) is quite significant. Since the younger generation tend to be more individualistic and less socially dependable, this dimension of national culture has shown to be a significant predictor of mindfulness for students with experience data. In construction work, emphasis is given to workers by their peers on the importance of teamwork and looking after one another.

#### 5.2.2. Uncertainty Avoidance

The results of this study show that this dimension of culture is a significant predictor of mindfulness for construction workers and the combined data. This dimension was also positively related to mindfulness for students with experience and novice students, even though it was not a significant predictor (see Table 7). This result implies that construction workers tend to avoid scenarios that cause ambiguity more than both student samples. Uncertainty avoidance measures the degree to which individuals respond to uncertain and ambiguous situations in the future [58,115,116]. Our study found a significant relationship between the uncertainty avoidance score of the construction workers’ dataset and their mindfulness scores. Lower scores of uncertainty avoidance signify tolerance and acceptance in the face of uncertain future and higher scores indicate the need to avoid uncertainty and/or taking unnecessary risk. Our finding implies that workers are aware of the dangers that they possibly face on the construction sites and are cognizant that uncertainty compromises their safety. Uncertainty avoidance is by far the strongest predictor of mindfulness score for construction workers in this study, and this dimension can be used by safety managers to assess workers risk taking behaviors as it relates to mindfulness.

## 6. Conclusions

High-risk organizations that operate in complex, high-hazard domains for extended periods of time have been using mindfulness to increase productivity and record better safety outcomes [44]. A growing number of studies are showing the benefits of using mindfulness to enhance safety and increased work performance in the workplace and suggesting that incorporating mindfulness can reduce incident occurrence. Unfortunately, limited was known regarding the extent to which personal characteristics of workers might impact their mindfulness state.

This paper examines ways to predict mindfulness of individual using relatively static factors—namely, by measuring personality traits and national culture. Using elastic net regression, the results show that certain personality traits and national cultural dimensions are associated with individuals’ dispositional mindfulness and the extent of influence of these variables can vary according to the levels of working experience in the construction industry. By detecting workers with lower mindfulness, the results of this study will enable safety managers to develop a more mindful workforce at construction sites by providing targeted training programs for workers with lower mindfulness or assigning those less mindful workers to activities that do not require higher levels of attention.

There are some limitations related to this study that are worth mentioning. First, the sample size of the study is limited to participants in the Northern Virginia region. Although the participants in the study came from a diverse background (workers and students consisted of Hispanic, black, and white), more data should be collected from diverse population of workers across the United States to generalize the results. Second, as limited number of variables are considered in the model, future studies should be conducted to investigate the role of other personal characteristics on mindfulness of construction workers. Third, potential sources of common method bias such as item characteristics, item context, or measurement context may affect the results of the findings [117], even though the variables passed the Harman’s single factor test (20.85% < 50%). Despite these limitations, this study contributes to the body of knowledge and have the potential to reduce cost of safety programs by enabling safety managers to provide personalized interventions for their workers according to their personal characteristics.

## Figures and Tables

**Table 1:** Definitions of the Hofstede’s cultural dimensions.

Dimension	Definition
Power distance	The way members of a group understand how authority is shared among them. Power distance measures the distribution of power in a society—in other words, this dimension of culture indicates the degree of inequality in which power is distributed to members of a society or organization [69].
Uncertainty avoidance (UA)	The way members of a group deal with unstructured situations and how comfortable they are dealing with such situations. Uncertainty avoidance measures a society’s susceptibility to uncertainty and ambiguity of future events or situations. High UA signifies not taking risks in an uncertain future while cultures with low UA have a greater tolerance and acceptance for events even when facing uncertainty [69].
Individualism and collectivism	The way members of a group consider acting towards someone outside their group. Individualism is the extent to which individuals should take care of themselves or their close ones and remain largely independent from other groups of the society. On the contrary, collectivism promotes the harmonious nature of society and prioritizes the interest of the whole group over the need of individual interest [69].
Masculinity and femininity	The way members of a group manifest their capabilities. Masculinity reflects the extent to which achievement, heroism, assertiveness, and material rewards are preferred and valued in a society, while femininity signifies compromise, cooperation, courtesy, and quality of life [69].
Long- and short-term orientation	The way members of a group feel toward future prize. Long-term orientation of cultural dimension signifies the value of persistence, perseverance, saving, and being able to adapt. On the other hand, short-term orientation reflects increased focus on the present or past and considers these factors more important than the future, value traditions, and social obligations [69].

**Table 2:** Penalization regression methods and associated penalties.

Method	Penalty
Ridge	β_j_^2^ (L_2_-penalty)
Lasso	|β_j_| (L_1_-penalty)
Elastic net	Combination of |β_j_| and β_j_^2^ (L_1_ and L_2_ penalty)

**Table 3:** Descriptive statistics of variables.

Variables	Mean			Median			Standard Deviation			Skewness			Kurtosis		
	ES	NS	W	ES	NS	W	ES	NS	W	ES	NS	W	ES	NS	W
Ext	27.3	25.6	26.8	28	26	27	5.2	6.2	3.77	−1.20	−0.05	−0.28	3.19	−0.77	−0.76
Agr	34.9	36.1	35.8	34	36	36	4.8	5.0	4.97	0.39	−0.58	−0.05	−0.18	0.37	−0.52
Con	36.0	35.4	38.3	36	36	39	5.1	5.2	5.45	0.09	−0.66	−1.86	−0.81	0.22	5.80
Neu	19.4	20.4	17.0	20	20	17.5	5.1	6.1	4.97	−0.31	0.41	−0.46	−0.36	−0.60	−0.29
Open	37.4	37.0	36.8	37	37	36.5	4.9	5.1	4.77	−0.24	0.15	0.11	−0.35	−0.26	0.63
PD	1.6	1.7	1.9	1.0	2.0	2.0	0.71	0.82	0.83	1.26	1.07	1.69	1.99	0.76	5.56
UA	4.6	4.5	4.3	5.0	5.0	4.0	0.50	0.57	0.87	−0.38	−1.06	−1.92	−1.96	2.42	5.83
IvsC	3.7	3.6	3.4	3.5	4.0	3.0	0.90	0.92	0.79	0.07	−0.72	0.36	−0.94	0.69	0.91
MvsF	2.4	1.8	2.2	2.5	1.0	2.0	1.20	1.03	1.16	0.62	1.15	0.39	−0.18	0.58	−1.29
MAAS	3.9	3.9	4.5	4.1	4.0	4.5	0.70	0.82	0.75	−0.74	−0.55	−0.42	0.47	0.65	0.32

ES = students with experience (*n* = 39), NS = novice students (*n* = 86), W = workers (*n* = 30). Ext = Extraversion, Agr = Agreeableness, Con = Conscientiousness, Neu = Neuroticism, Open = Openness, PD = Power Distance, UA = Uncertainty Avoidance, IvsC = Individualism vs. Collectivism, MvsF = Masculinity vs. Femininity, MAAS = Mindfulness Score.

**Table 4:** Correlation matrix of variables.

Variables	Ext			Agr			Cons			Neu			Open		
	ES	NS	W	ES	NS	W	ES	NS	W	ES	NS	W	ES	NS	W
Ext	1.00	1.00	1.00												
Agr	0.10	0.22 **	0.12	1.00	1.00	1.00									
Con	0.33 **	0.23 **	0.01	0.24 **	0.29 **	0.52 ***	1.00	1.00	1.00						
Neu	0.34 ***	0.40 ***	0.08	0.27 **	0.22 **	0.42 ***	0.47 ***	0.21 *	0.44 ***	1.00	1.00	1.00			
Open	0.07	0.23	0.29 **	0.26 **	0.16	0.31 **	0.16	0.22 *	0.20 *	0.01	0.10	0.07	1.00	1.00	1.00
PD	0.04	0.12	0.22	0.11	0.21 *	0.25 **	0.16	0.11	0.25 **	0.06	0.14	0.23 *	0.03	0.06	0.18
UA	0.12	0.29 **	0.09	0.08	0.16	0.39 ***	0.01	0.08	0.31 **	0.14	0.14	0.08	0.21 *	0.24 **	0.27 *
IvsC	0.14	0.17	0.09	0.22 **	0.12	0.14	0.21 *	0.08	0.21 *	0.36 ***	0.21 **	0.23 *	0.10	0.02	0.02
MvsF	0.19 *	0.07	0.32 ***	0.33 ***	0.17 *	0.10	0.06	0.13	0.16	0.11	0.08	0.15	0.13	0.14	0.12
MAAS	0.28 **	0.16	0.14	0.15 *	0.25 **	0.21 **	0.31 **	0.21 *	0.47 ***	0.33 ***	0.22 **	0.29 **	0.08	0.01	0.03
**Variables**	**PD**			**UA**			**IvsC**			**MvsF**			**MAAS**		
	**ES**	**NS**	**W**	**ES**	**NS**	**W**	**ES**	**NS**	**W**	**ES**	**NS**	**W**	**ES**	**NS**	**W**
Ext															
Agr															
Con															
Neu															
Open															
PD	1.00	1.00	1.00												
UA	0.05	0.07	0.07	1.00	1.00	1.00									
IvsC	0.30 **	0.01	0.33 ***	0.09	0.23 *	0.06	1.00	1.00	1.00						
MvsF	0.38 ***	0.28 **	0.24 **	0.15	0.05	0.10	0.15	0.15	0.21 *	1.00	1.00	1.00			
MAAS	0.01	0.13	0.13	0.06	0.07	0.40 ***	0.08	0.01	0.04	0.14	0.13	0.09	1.00	1.00	1.00

ES = students with experience (*n* = 39), NS = novice students (*n* = 86), W = workers (*n* = 30). Ext = Extraversion, Agr = agreeableness, Con = Conscientiousness, Neu = Neuroticism, Open = Openness, PD = Power Distance, UA = Uncertainty Avoidance, IvsC = Individualism vs. Collectivism, MvsF = Masculinity vs. Femininity, MAAS = Mindfulness Score. * *p* < 0.1 ** *p* < 0.05, *** *p* < 0.01. Underlined correlation = negative correlation

**Table 5:** Number of items and Cronbach alpha for all scales.

Scales	Number of Items	Cronbach’s Alpha
Extraversion	8	0.81
Agreeableness	9	0.71
Conscientiousness	9	0.78
Neuroticism	8	0.79
Openness	10	0.70
Power distance	5	0.73
Uncertainty avoidance	5	0.82
Individualism vs. collectivism	6	0.78
Masculinity vs. femininity	4	0.83
MAAS	15	0.88

**Table 6:** Range of alpha values and corresponding parameter estimates and standard errors.

	Students with Experience (*n* = 39)		Novice Students (*n* = 84)		Workers (*n* = 30)	
Alpha Value	Estimate	Std Error	Estimate	Std Error	Estimate	Std Error
0.10	0.596	0.077	0.670	0.064	0.378	0.069
0.25	0.556	0.078	0.668	0.058	0.399	0.067
0.50	0.587	0.065	0.663	0.067	0.414	0.059
0.75	0.549	0.073	0.686	0.065	0.423	0.052
0.90	0.588	0.079	0.652	0.061	0.434	0.056

**Table 7:** Parameter estimates of predictors, Wald statistics, 95% confidence interval and variance inflation factor.

Terms	Estimate			Std Error			Wald Chi Square			Prob > Chi Square			Lower 95%			Upper 95%			VIF		
	ES	NS	W	ES	NS	W	ES	NS	W	ES	NS	W	ES	NS	W	ES	NS	W	ES	NS	W
Int	5.38	4.00	0.44	1.76	1.13	1.42	9.31	12.47	0.09	<0.01 **	<0.01 **	0.76	1.92	1.78	−2.35	8.84	6.23	3.22	0	0	0
Ext	0	0	0	0	0	0	0	0	0	1.00	1.00	1.00	0	0	0	0	0	0	0	0	0
Agr	0	0.03	−0.03	0	0.01	0.03	0	5.06	1.44	1.00	0.02 *	0.23	0	0.00	−0.09	0	0.06	0.02	0	0.93	3.44
Con	0.01	0.01	0.10	0.03	0.02	0.02	0.02	0.18	16.06	0.90	0.67	<0.01 **	−0.05	−0.03	0.05	0.06	0.04	0.15	2.63	1.55	3.04
Neu	−0.08	−0.04	−0.01	0.03	0.02	0.02	4.88	5.45	0.09	0.03 *	0.02 *	0.76	−0.14	−0.08	−0.06	−0.01	−0.01	0.04	3.50	2.25	2.50
Open	0	−0.01	0	0	0.02	0	0	0.39	0	1.00	0.53	1.00	0	−0.05	0	0	0.03	0	0	1.82	0
PD	0	0	−0.01	0	0	0.14	0	0	0.00	1.00	1.00	0.94	0	0	−0.29	0	0	0.27	0	0	2.13
UA	0.17	0.05	0.34	0.22	0.13	0.10	0.60	0.17	12.38	0.44	0.68	<0.01 **	−0.27	−0.20	0.15	0.61	0.32	0.53	1.39	1.03	1.12
IvsC	−0.24	−0.09	0.11	0.11	0.10	0.14	4.60	0.91	0.55	0.03 *	0.34	0.46	−0.47	−0.27	−0.18	−0.02	0.09	0.39	1.18	1.22	2.04
MvsF	0	0	0	0	0	0	0	0	0	1.00	1.00	1.00	0	0	0	0	0	0	0	0	0

* *p* < 0.05, ** *p* < 0.01. ES = students with experience (*n* = 39), NS = Novice students (*n* = 84), W = workers (*n* = 30) Int =intercept, Ext = Extraversion, Agr = agreeableness, Con = Conscientiousness, Neu = Neuroticism, Open = Openness, PD = Power Distance, UA = Uncertainty Avoidance, IvsC = Individualism vs. Collectivism, MvsF = Masculinity vs. Femininity.

## Data Availability

All data, models, or code generated or used during the study are available from the corresponding author by request.
